# Less Is More: Standardization and Description of Partial Transversus Abdominis Release (pTAR) for W2–W3 Midline Incisional Hernia Repair

**DOI:** 10.3390/jcm15197600

**Published:** 2026-09-30

**Authors:** Umberto Bracale, Biancamaria Iacone, Antonio Gargiulo, Anna Tedesco, Maria De Falco, Salvatore Tramontano, Marta Cavalli, Gianpiero Campanelli

**Affiliations:** 1 Department of Medicine, Surgery and Dentistry “Scuola Medica Salernitana”, University of Salerno, AOU San Giovanni di Dio e Ruggi d’Aragona, 84084 Salerno, Italy; umbertobracale@gmail.com (U.B.); biancamariaiacone@gmail.com (B.I.); anna.mirea95@libero.it (A.T.); mariadefalco97@gmail.com (M.D.F.); salvytra@libero.it (S.T.); 2 I.R.C.C.S. Ospedale Galeazzi-Sant’Ambrogio, University of Insubria, 20157 Milan, Italy; marta_cavalli@hotmail.it (M.C.); giampiero.campanelli@grupposandonato.it (G.C.)

**Keywords:** ventral hernia, incisional hernia, transversus abdominis release, partial TAR, tailored TAR, posterior component separation, retromuscular mesh, Rives-Stoppa repair, abdominal wall reconstruction

## Abstract

**Background:** Posterior component separation with transversus abdominis release (TAR) enables tension-free reconstruction of large midline incisional hernias but, when performed as a complete bilateral release, may expose selected patients to extensive lateral dissection and related morbidity. In daily practice, many surgeons already limit the release to the segment that is strictly required (“tailored”, “limited” or “modified” TAR), but this conservative use of TAR has never been explicitly defined, illustrated or reported in a reproducible way. We define this approach as partial transversus abdominis release (pTAR) and describe it in a standardized, stepwise fashion, reporting the preliminary outcomes of an initial single-surgeon series. **Methods:** We retrospectively reviewed all patients who underwent open midline incisional hernia repair with retromuscular synthetic mesh and pTAR performed by a single surgeon at a single center between January 2024 and January 2025. Complex hernias were defined a priori. Continuous variables are reported as mean ± standard deviation. The report is a technical note and preliminary case series. **Results:** Ten patients with complex W2–W3 midline incisional hernias were included. pTAR was performed in the subxiphoid segment in eight cases and in the periumbilical/middle segment in two cases. No major intraoperative complications occurred. Two patients developed a postoperative seroma, both classified as surgical site occurrences (SSO) not requiring intervention. One recurrence and one readmission were recorded; no reoperation or death occurred. **Conclusions:** pTAR is presented as a reproducible, standardized variant of posterior component separation rather than as a novel operation or as proof of superiority over complete TAR. Since this is a small, retrospective, single-surgeon, single-center series with a follow-up shorter than 12 months, the data demonstrate feasibility only; the hypothesized tissue-sparing advantage must be tested in prospective, registered, comparative studies.

## 1. Introduction

Incisional hernia is one of the most frequent complications of abdominal surgery, developing after a substantial proportion of midline laparotomies and representing a major source of morbidity, impaired quality of life and health-care cost [1]. Surgical management has evolved considerably over the last three decades. Ramirez et al. introduced the anterior component separation technique, mobilizing the myofascial units of the abdominal wall to achieve midline closure [2]. The Rives-Stoppa repair subsequently established the retromuscular plane, in contact with the posterior rectus sheath, as a biomechanically favorable position for mesh placement [3]. However, classical retromuscular dissection is limited laterally by the linea semilunaris and may be insufficient for wide (W3) defects or hernias with loss of domain [4].

In 2012, Novitsky et al. described posterior component separation with transversus abdominis release (TAR), extending the retromuscular dissection by dividing the transversus abdominis muscle medial to the linea semilunaris and developing a wide retromuscular/preperitoneal space [5]. TAR allows extensive medial advancement of the posterior rectus sheath and tension-free closure even in large defects. Nonetheless, complete TAR is not free from complications: pooled analyses of open TAR report surgical site occurrence (SSO) rates of approximately 21.7% and surgical site infection (SSI) rates of approximately 9.1% [6], and inappropriate or excessive lateral dissection may injure the segmental neurovascular bundles, leading to rectus denervation, abdominal-wall laxity or iatrogenic lateral hernia [5,7]. For these reasons, the indications for, and limits of, TAR remain actively debated [7,8].

Importantly, the principle that the extent of the transversus release should be proportionate to the defect—and that a complete bilateral release is not always necessary—is already applied intuitively by many abdominal-wall surgeons under heterogeneous and largely informal labels such as “tailored”, “limited” or “modified” TAR [7,9]. To date, however, this conservative use of posterior component separation has neither been given a consistent definition nor been described, illustrated and reported in a reproducible manner. The aim of this Technical Note is therefore not to claim a new operation, but to provide a clear definition, a standardized description with intraoperative and schematic documentation, and explicit selection criteria for what we term partial transversus abdominis release (pTAR). This preliminary case series should be interpreted as an early clinical experience intended to support the feasibility and reproducibility of the technique, rather than as evidence of superiority over complete TAR.

## 2. Materials and Methods

### 2.1. Study Design, Patients and Definitions

We performed a retrospective analysis of a prospectively maintained clinical record of all patients who underwent open midline incisional hernia repair with retromuscular synthetic mesh and pTAR, performed by a single surgeon (U.B.) at a single center between January 2024 and January 2025. Hernias were classified according to the European Hernia Society (EHS) classification of primary and incisional abdominal-wall hernias [4].

For the purpose of this study, a hernia was defined as complex when it presented at least one of the following features: EHS width class W2 (4–10 cm) or W3 (≥10 cm); a recurrent defect; loss of domain; a previously contaminated or multiple-operated abdomen; or an anticipated need for a component-separation procedure to achieve tension-free midline closure. Inclusion required a midline W2–W3 incisional hernia treated with retromuscular mesh and pTAR. Patients undergoing complete bilateral TAR, bridged repairs or non-midline reconstructions were excluded.

### 2.2. Definition of pTAR, Indications and Difference from Complete TAR

**Figure 1:**
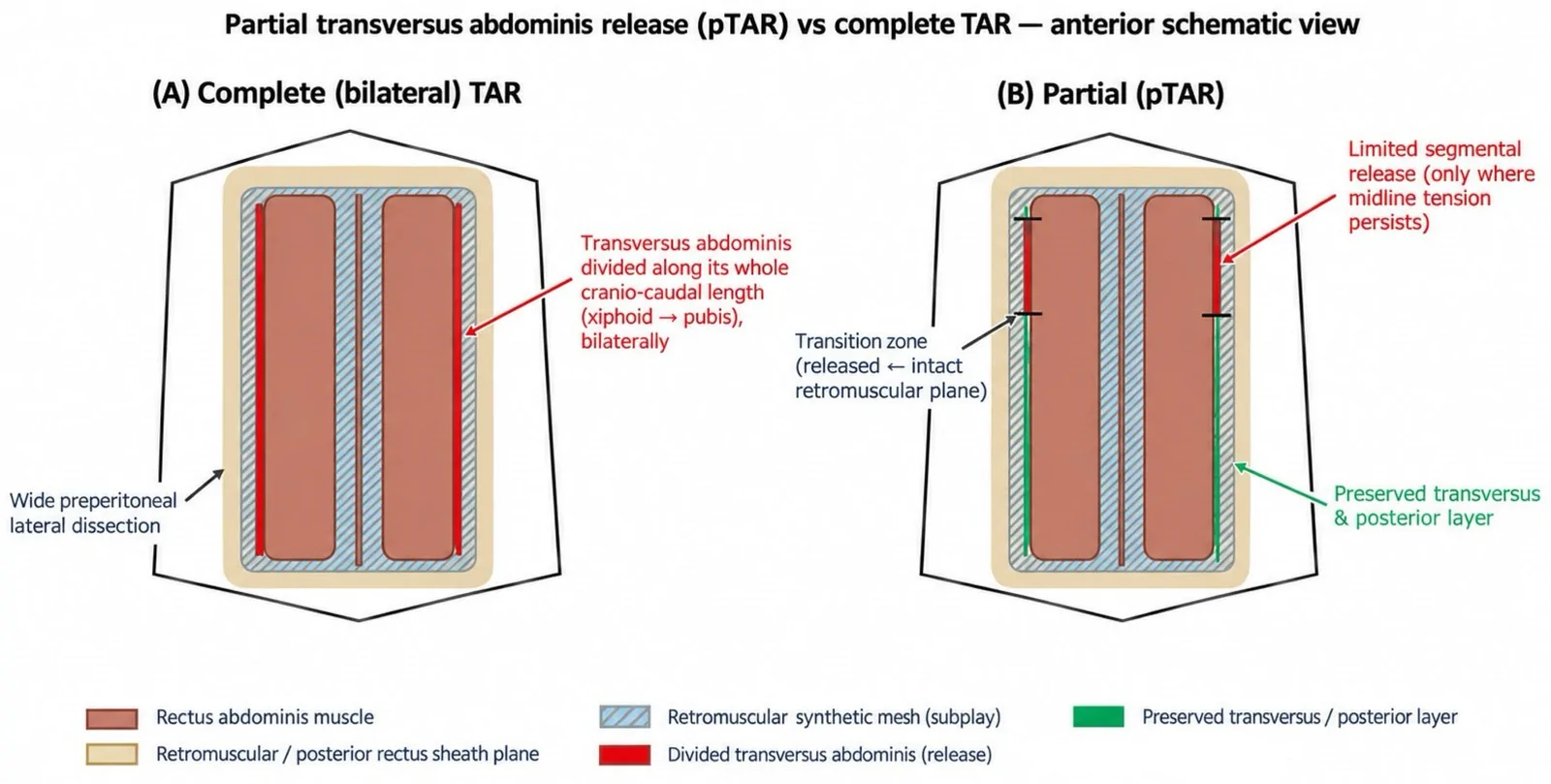
Schematic anterior view of the abdominal wall comparing complete (bilateral) transversus abdominis release (**A**) with partial TAR (pTAR) (**B**). In complete TAR, the transversus is divided along its whole cranio–caudal length bilaterally, with wide preperitoneal dissection. In pTAR the transversus and posterior layer are preserved except over the limited segment where midline tension persists; the transition zones between the released and the intact retromuscular plane are indicated. The retromuscular synthetic mesh is placed in the same plane in both techniques.

Partial transversus abdominis release (pTAR) is defined as the division of the transversus abdominis muscle limited to the cranio–caudal segment in which tension-free midline restoration cannot otherwise be achieved, leaving the remainder of the transversus and of the posterior rectus sheath intact. It differs from the complete TAR described by Novitsky et al., in which the transversus is divided from the subxiphoid region to the pubis, usually bilaterally [5]. Conceptually, pTAR is the formalization of the “tailored/limited” TAR already used in practice (Figure 1).

Candidate patients were identified preoperatively by clinical examination and computed tomography (CT). Published CT morphometric parameters associated with the need for a component-separation procedure—such as increasing defect width and defect area, and unfavorable rectus defect ratio (RDR)—were used to anticipate which patients might require a release [10,11,12]. In hernias with loss of domain, the Tanaka index (hernia-sac to abdominal-cavity volume ratio) was used to guide preoperative optimization [13]. The final decision to perform pTAR, and the extent of the release, were taken intraoperatively when tension-free midline approximation could not be obtained in a specific segment after complete retromuscular dissection. The predominance of subxiphoid releases is supported by the anatomical observation that the transversus abdominis extends within the rectus sheath at the costal margin, where the standard retrorectus plane is shortest [14].

### 2.3. Surgical Technique

**Figure 2:**
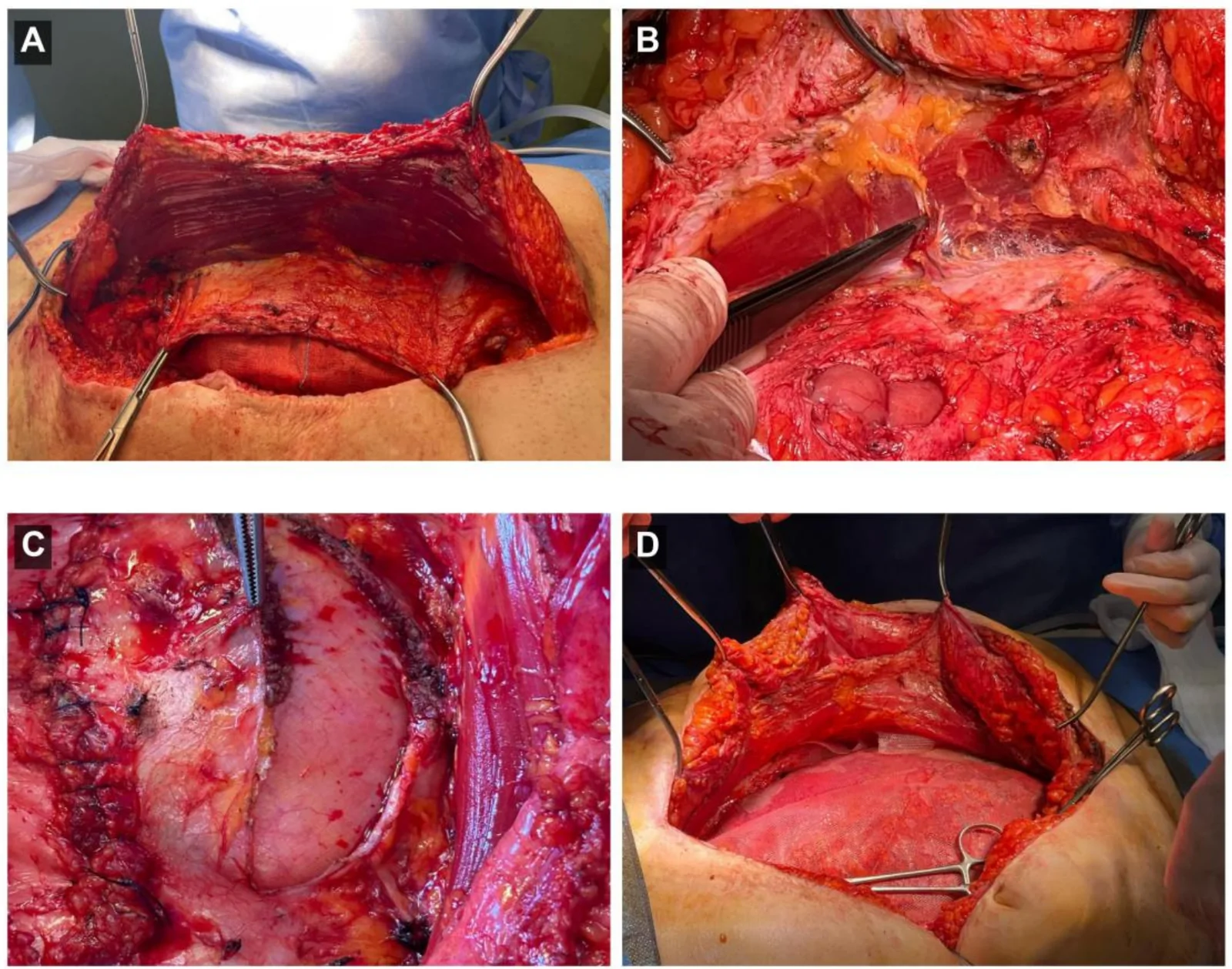
Intraoperative steps of pTAR. (**A**) Development of the retromuscular plane with medial retraction of the rectus abdominis muscle. (**B**) Limited segmental division of the transversus abdominis exposing the preperitoneal plane. (**C**) Transition zone between the released segment and the intact posterior rectus sheath. (**D**) Shaped synthetic mesh positioned in the retromuscular plane before anterior fascial closure.

The procedure begins with midline laparotomy, access to the abdominal cavity and meticulous adhesiolysis. The retromuscular plane is developed from the medial edge of the rectus abdominis toward the linea semilunaris, preserving the neurovascular bundles and inferior epigastric vessels, and is extended cranially to the subxiphoid space and caudally into the Retzius space, exposing the pubic symphysis and both Cooper ligaments. When tension-free midline restoration cannot be achieved in a specific segment, pTAR is performed: following the principles of posterior component separation, the posterior rectus sheath is incised approximately 0.5–1 cm medial to the linea semilunaris and the transversus abdominis is divided only along the segment required to complete midline approximation in that limited area (Figure 2A,B). Particular attention is paid to the transition zone between the released segment—where the preperitoneal plane is directly exposed—and the adjacent intact retromuscular plane, in order to avoid mesh folding and to reinforce this potential weak point (Figure 2C).

A shaped macroporous monofilament polypropylene synthetic mesh is positioned in the retromuscular space as a sublay and fixed with monofilament sutures according to Rives-Stoppa principles (Figure 2D). The posterior layer is closed to exclude the mesh from the abdominal viscera. The anterior fascia/linea alba is reconstructed ventral to the mesh using a continuous small-bites technique with a slowly absorbable monofilament suture, in line with current closure guidelines [15,16]. Two suction drains are routinely positioned, one above the mesh and one in the subcutaneous plane; drains were removed when daily output fell below 50 mL/24 h. In two patients, abdominal-wall traction with a Fasciotens device (Fasciotens GmbH, Cologne, Germany) was applied before midline restoration to facilitate reapproximation of the anterior component; because this device independently influences fascial mobilization, these two cases are reported separately and are considered a potential confounder.

### 2.4. Follow-Up

The follow-up protocol consisted of clinical examination and ultrasound assessment at 3 and 12 months. Recurrence and postoperative complications were recorded throughout the available follow-up period. Because patients were enrolled progressively between January 2024 and January 2025, not all patients could have reached the 12-month assessment at the time of this analysis; the actual follow-up is therefore reported as the mean (±standard deviation) per patient rather than as fixed cohort time points, and the 3- and 12-month assessments should be read as protocol time points rather than as a completed cohort-wide evaluation. The actual mean follow-up was 7.6 ± 3.9 months (range 2–13).

### 2.5. Outcomes and Definitions

Outcomes included operative time, intraoperative complications, SSI, SSO, postoperative seroma, length of hospital stay, readmission, reoperation, recurrence during follow-up and mortality. Seroma was recorded within the SSO category; in this series the two seromas correspond to the two SSO events, with no SSI. Postoperative complications were graded according to the Clavien-Dindo classification [17]. Recurrence was defined as a clinically and/or ultrasonographically detected fascial defect at the repair site during follow-up.

### 2.6. Statistical Analysis

Given the small sample, continuous variables are summarized as mean ± standard deviation and categorical variables as counts and percentages. Because the series is exploratory, no inferential comparison was performed.

## 3. Results

Ten patients with complex W2–W3 midline incisional hernias were treated with pTAR between January 2024 and January 2025 (four women, six men). Baseline demographic and clinical characteristics are detailed in Table 1; comorbidities are reported in full in that table and are not restated selectively here, to ensure consistency between the text and the table. According to the EHS classification, all included patients presented W2 or W3 midline defects [4]; operative and defect characteristics are reported in Table 2.

Operative and defect characteristics are summarized in Table 2. pTAR was performed in the subxiphoid segment in eight patients and in the periumbilical/middle segment in two patients; the release was bilateral in eight patients and unilateral in two. A Fasciotens device was used in two patients. The mean operative time was 120 ± 30 min.

Postoperative outcomes are reported in Table 3. No major intraoperative complications occurred. Two patients (20%) developed a postoperative seroma, both classified as SSO (Clavien-Dindo grade I) and managed conservatively; no SSI was observed. The mean length of hospital stay was 5 ± 2 days. One readmission (10%) occurred during the follow-up period and was managed without reoperation. One recurrence (10%) was detected on clinical and ultrasound follow-up twelve months after the surgery; it was managed conservatively and did not require reoperation during the study period. No reoperation and no mortality were recorded. In the subgroup of patients treated with Fasciotens, no postoperative complications or adverse events were observed during the follow-up period. Both suprafascial and subcutaneous drains were used in all patients and removed once daily output fell below the 50 mL/24 h threshold described above.

## 4. Discussion

The optimal strategy for ventral incisional hernia repair remains debated, and the 2023 European Hernia Society guidelines recommend individualizing the approach according to patient and hernia characteristics and surgeon expertise, while strongly supporting retromuscular mesh placement [18]. Within this framework, the present Technical Note does not propose a new operation, but its goal is to define, standardize and visually document a conservative, segment-limited use of posterior component separation that is already practised informally [19]. Within the IDEAL framework for evaluating surgical innovation, this report corresponds to an IDEAL Stage 1–2a (Idea/Development) description of pTAR, intended to precede a Stage 2b/3 comparative evaluation against complete TAR [19].

This positioning directly addresses the principal limitation of the concept: as noted in the literature, the idea that the extent of the transversus release should be proportionate to the defect is not new. Zolin et al. explicitly examined the real indications and limits of TAR [7], and Riediger and Köckerling recently discussed borderline situations in which TAR alone is insufficient and bridging or alternative strategies are considered [8]; segment-tailored releases are also implicit in earlier technical reports [9]. The value of pTAR therefore lies not in novelty of principle but in providing a reproducible definition, an illustrated description and explicit, CT-informed selection criteria, which to our knowledge have not previously been reported together.

Complete open TAR is an effective but not innocuous procedure. In a single-center analysis of 1203 patients, Zolin et al. reported a composite hernia-recurrence rate of 26% at a median follow-up of 2 years (interquartile range 1–4 years), with successful fascial reapproximation in 92% and measurable improvements in hernia-specific quality of life regardless of recurrence [20]. Pooled data from open TAR similarly show clinically relevant SSO (≈21.7%) and SSI (≈9.1%) rates [6]; short-term morbidity also compares the surgical access route between open and robotic [21]. These figures define the morbidity baseline of complete TAR and provide the rationale for exploring whether a more limited release is sufficient in selected patients, which is a question that the present uncontrolled series cannot answer and that we frame explicitly as a hypothesis.

Patient selection was informed by preoperative CT. Rather than relying on isolated, unsourced numerical thresholds, we used the morphometric parameters that have been associated with the need for a component separation in published series such as RDR, Component Separation Index (CSI) and Tanaka Index [10,11,12,13]. We recommend that these parameters can be a guide to the selection of the best candidate for the execution of pTAR, although the final decision is made intraoperatively, because these parameters cannot reliably predict technical feasibility.

Several limitations must temper the interpretation of these data and are stated explicitly. First, the claim that pTAR reduces unnecessary lateral dissection and spares tissue is inherently comparative, yet there was no complete TAR control group, no matched cohort and no independent quantification of the dissected area. With ten uncontrolled cases, the series can demonstrate feasibility but cannot establish lower morbidity or less dissection than complete TAR; all such statements are therefore presented as hypotheses to be tested. Second, the “less is more” message requires clarification: because the release was bilateral in 80% of cases, the main difference from a standard bilateral TAR is the cranio–caudal length of muscle divided rather than its laterality. The preserved transversus length relative to a complete release is the key of pTAR, and it is important to note this message in order to avoid unnecessary dissections. Third, a Fasciotens device was used in 20% of patients; because it independently affects fascial mobilization, the outcomes in those cases cannot be attributed to pTAR alone, and they represent a confounding co-intervention. Fourth, the decision to release remained, in part, an intraoperative judgement guided by direct visual assessment of tension-free midline approximation rather than by a standardized intraoperative measurement; the threshold for release is not yet fully objective. Finally, the series is small, retrospective, single-surgeon and single-center, with a mean follow-up shorter than 12 months and without an imaging-based assessment beyond 12 months, so it cannot support conclusions on long-term recurrence or function.

A 10% recurrence in a series of ten patients is not negligible and is reported consistently across the abstract, results, tables and conclusions. The single recurrence was managed conservatively, without reoperation during the study period. Such an event rate, in the context of complex W2–W3 defects, underscores that pTAR is offered as a feasible technical option and not as a means of improving durability.

Future directions point naturally toward formal prospective clinical evaluation of the technique. The appropriate next step is a prospective, registered study with consecutive enrollment, standardized CT morphometry, objective intraoperative measurement of the released segment, Clavien-Dindo grading and a direct comparison with complete TAR [7,19]. Such a design would allow the hypothesized tissue-sparing benefit to be tested rather than assumed and would define which patients benefit most from a partial release.

## 5. Conclusions

pTAR is a standardized, reproducible variant of posterior component separation in which the transversus abdominis is divided only in the segment where additional medial advancement is required, leaving the remainder of the muscle and posterior layer intact. In this preliminary single-surgeon series, it appeared feasible and was associated with low early morbidity and one recurrence during a follow-up shorter than 12 months. Its main potential advantage—a reduction in unnecessary lateral dissection while preserving tension-free midline restoration—remains a hypothesis. Prospective, registered, comparative studies with larger samples, standardized CT-based assessment and longer follow-up are required before any clinical advantage over complete TAR can be claimed.

## Figures and Tables

**Table 1:** Baseline demographic and clinical characteristics (n = 10).

Age (years), mean ± SD	53.3 ± 13.6
BMI (kg/m^2^), mean ± SD	26.3 ± 6.5
Sex, male/female	6 (60%)/4 (40%)
ASA class I–II/III–IV ^1^	3 (30%)/7 (70%)
Recurrent hernia	1 (10%)
History of cardiovascular disease ^2^	6 (60%)
Smoking	4 (40%)
Diabetes mellitus	3 (30%)

^1^ ASA Physical Status assigned by the attending anesthesiologist. ^2^ treated arterial hypertension, ischemic heart disease, arhythmia or heart failure.

**Table 2:** Defect and operative characteristics (n = 10).

EHS Width Class	W2–W3 (Complex Midline Defects)
Defect width (cm), mean ± SD	10 ± 2
Defect length (cm), mean ± SD	12 ± 3
Mesh width (cm), mean ± SD	27 ± 3
Mesh length (cm), mean ± SD	35 ± 5
pTAR location: subxiphoid/periumbilical	8 (80%)/2 (20%)
pTAR laterality: bilateral/unilateral	8 (80%)/2 (20%)
Fasciotens device used	2 (20%)
Operative time (min), mean ± SD	120 ± 30

**Table 3:** Postoperative outcomes (n = 10).

Major intraoperative complications	0 (0%)
Surgical site infection (SSI)	0 (0%)
Surgical site occurrence (SSO)—seroma	2 (20%)
Clavien-Dindo grade of complications	I
Length of hospital stay (days), mean ± SD	5 ± 2
Readmission	1 (10%)
Recurrence during follow-up	1 (10%)
Reoperation	0 (0%)
Mortality	0 (0%)
Actual follow-up (months), mean ± SD	7.6 ± 3.9 (range 2–13)

Data are n (%) or mean ± SD. SSI, surgical site infection; SSO, surgical site occurrence. The two seromas are the two SSO events; there were no SSI. Because enrollment was staggered between January 2024 and January 2025, not every patient had reached the 12-month protocol assessment at the time of analysis; actual per-patient follow-up is reported instead of the fixed 3- and 12-month time points.

## Data Availability

The data presented in this study are available on reasonable request from the corresponding author. The data are not publicly available because of privacy and ethical restrictions relating to identifiable surgical patients.
